# Supplementary material for: Actuation and Mapping of Surface Acoustic Wave Induced High-Frequency Wavefields on Suspended Graphene Membranes
Source: ACS Nano. 2025 Apr 1;19(14):14044–52. doi: 10.1021/acsnano.4c18508 (PMC12004930; doi:10.1021/acsnano.4c18508)
Supplement: Supplementary file 1 — nn4c18508_si_001.pdf [file nn4c18508_si_001.pdf]

Supporting Information

Actuation and mapping of surface acoustic wave induced high-frequency wavefields on suspended graphene membranes

Hande N. Açıkgöz[1], Dong Hoon Shin[2], Inge C. van der Knijff[1], Allard J. Katan[3], Xiliang Yang[1], Peter G. Steeneken[1], Gerard J. Verbiest[1,], Sabina Caneva[1,*]*

[1]Department of Precision and Microsystems Engineering, Delft University of Technology, Mekelweg 2, 2628 CD Delft, The Netherlands

[2]Department of Electronics and Information Engineering, Korea University, Sejong 30019, Republic of Korea

[3]Kavli Institute of Nanoscience Delft, Lorentzweg 1, 2628 CJ Delft, The Netherlands

*E-mail: s.caneva@tudelft.nl

g.j.verbiest@tudelft.nl

Content:

S1.1 AFAM imaging at different locations in the SAW delay line

S1.2 Microcavity profile and SAW field across a cavity

S1.3 AFAM Measurements of devices D2 and D5

S1.4 AFAM Measurements at different frequencies

S2.1 Device data: D1

S2.2 Device data: D2

S2.3 Device data: D3

S2.4 Device data: D4

S2.5 Device data: D5

S3 AFAM data processing

S4 Dispersion curve fit

S5 COMSOL Simulation

S6 Additional cavity geometries

S7 LDV vs AFAM for mapping suspended graphene vibrations

S8.1 AFM topography scans across features

S8.2 2D material transfer with PDMS dome stamp

S1.1 AFAM imaging at different locations in the SAW delay line

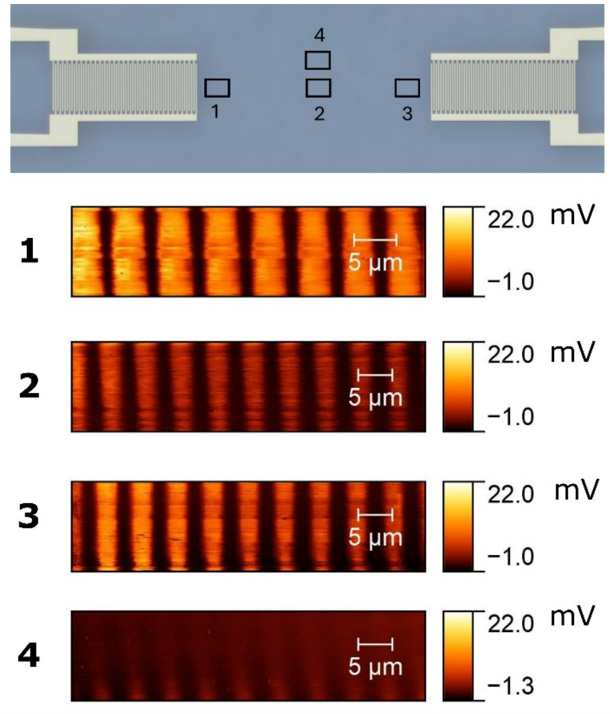

Figure S1.1. AFAM scans at different locations on the delay line.

S1.2 Microcavity profile and SAW field across a cavity

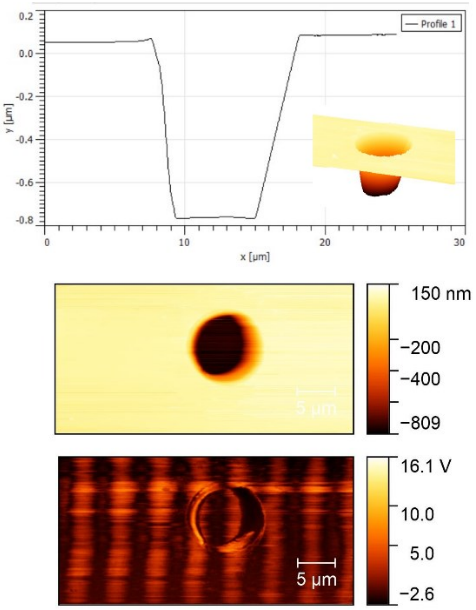

Figure S1.2. Microcavity topography and AFAM scan across the microcavity.

S1.3 AFAM Measurements of devices D2 and D5

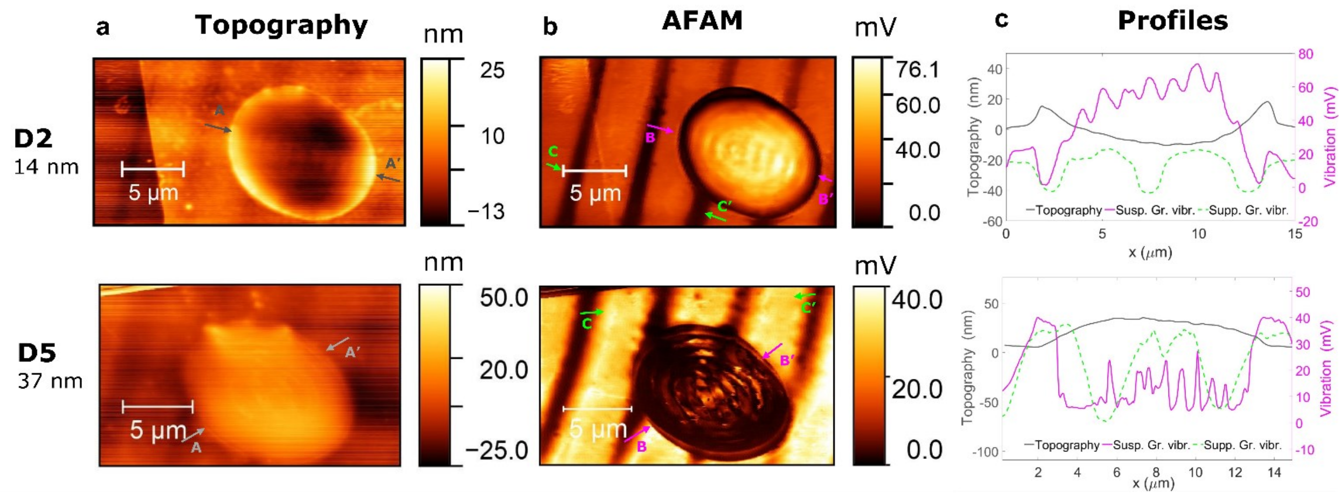

Figure S1.3. Topography and vibration field mapping of devices D2 and D5.

S1.4 AFAM Measurements at different frequencies

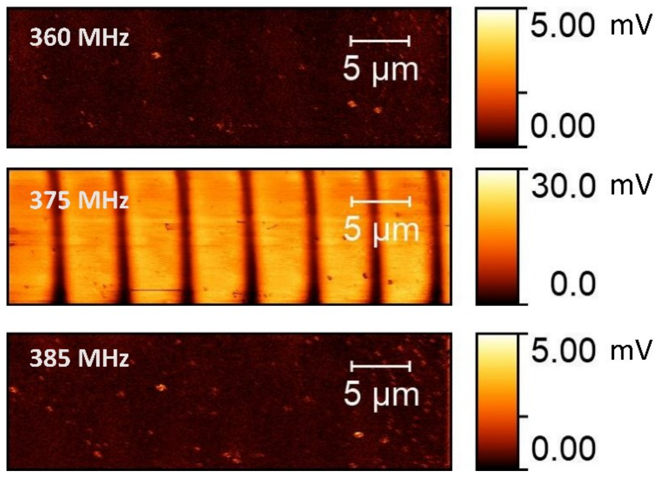

Figure S1.4. AFAM mapping of SAW field on bare substrate at different frequencies.

S2.1 Device data: D1

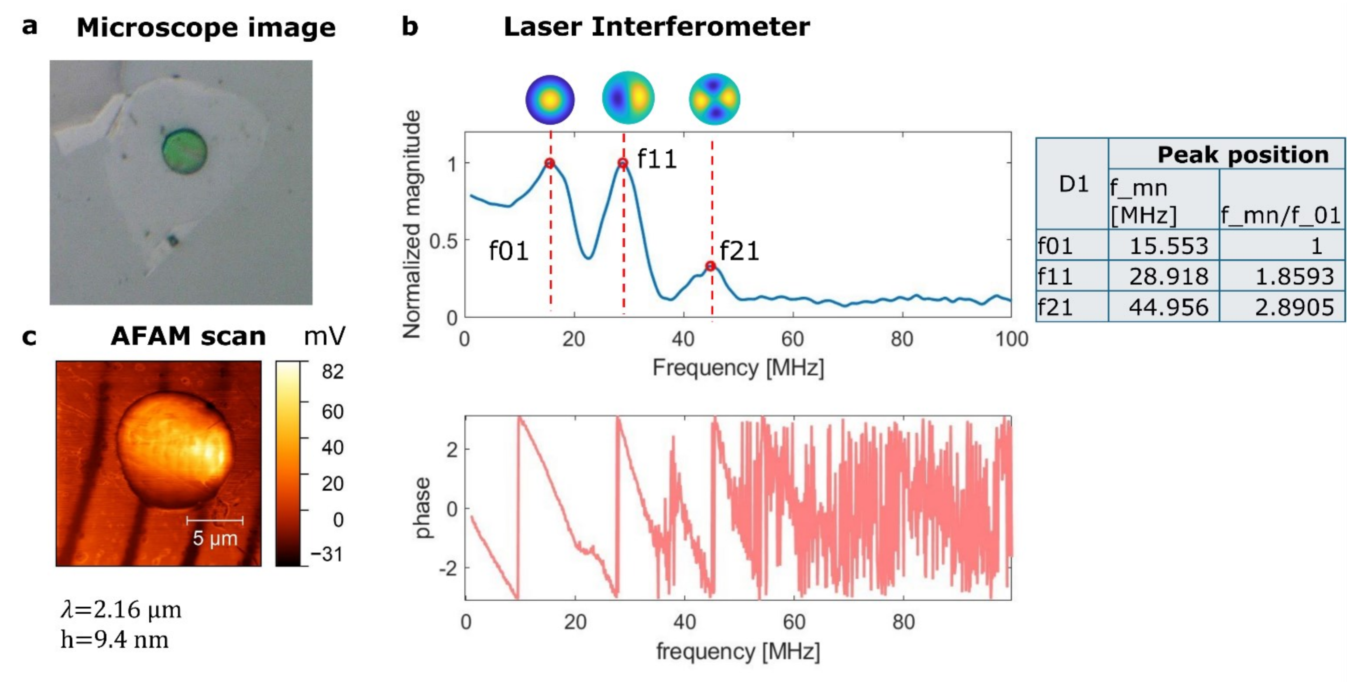

Figure S2.1. Data for Device 1. a) Microscope image, b) Laser interferometer measurements, c) AFAM scan.

S2.2 Device data: D2

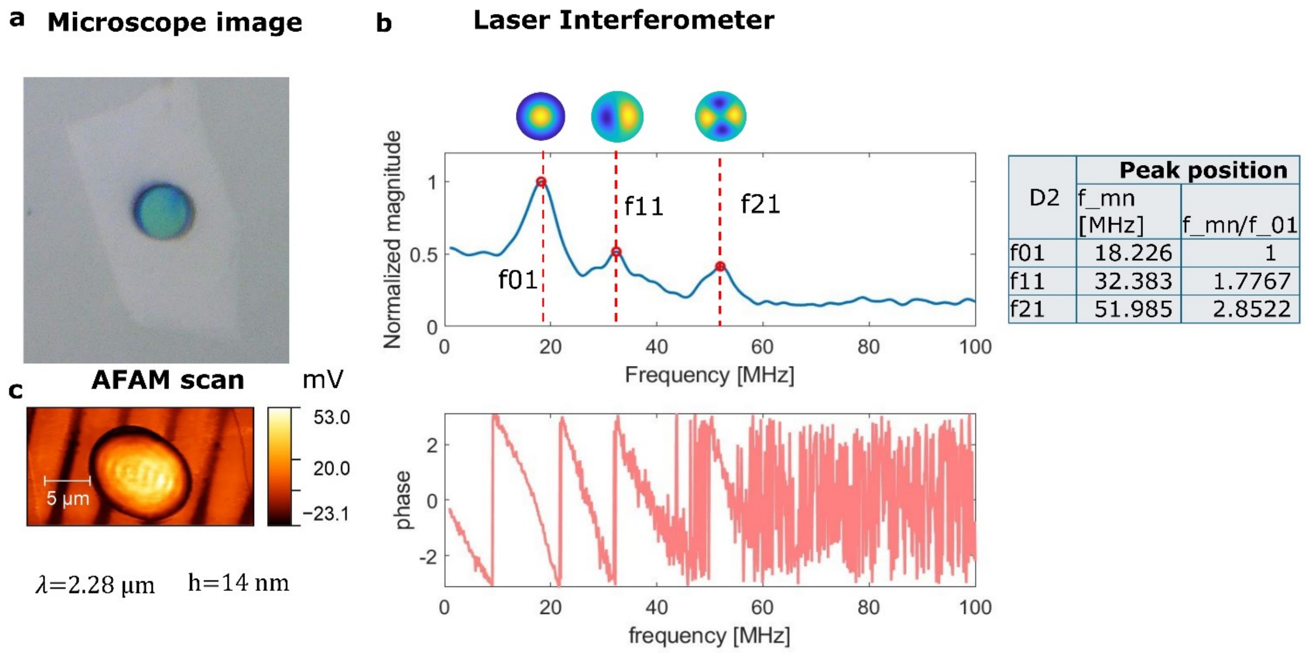

Figure S2.2. Data for Device 2. a) Microscope image, b) Laser interferometer measurements, c) AFAM scan.

S2.3 Device data: D3

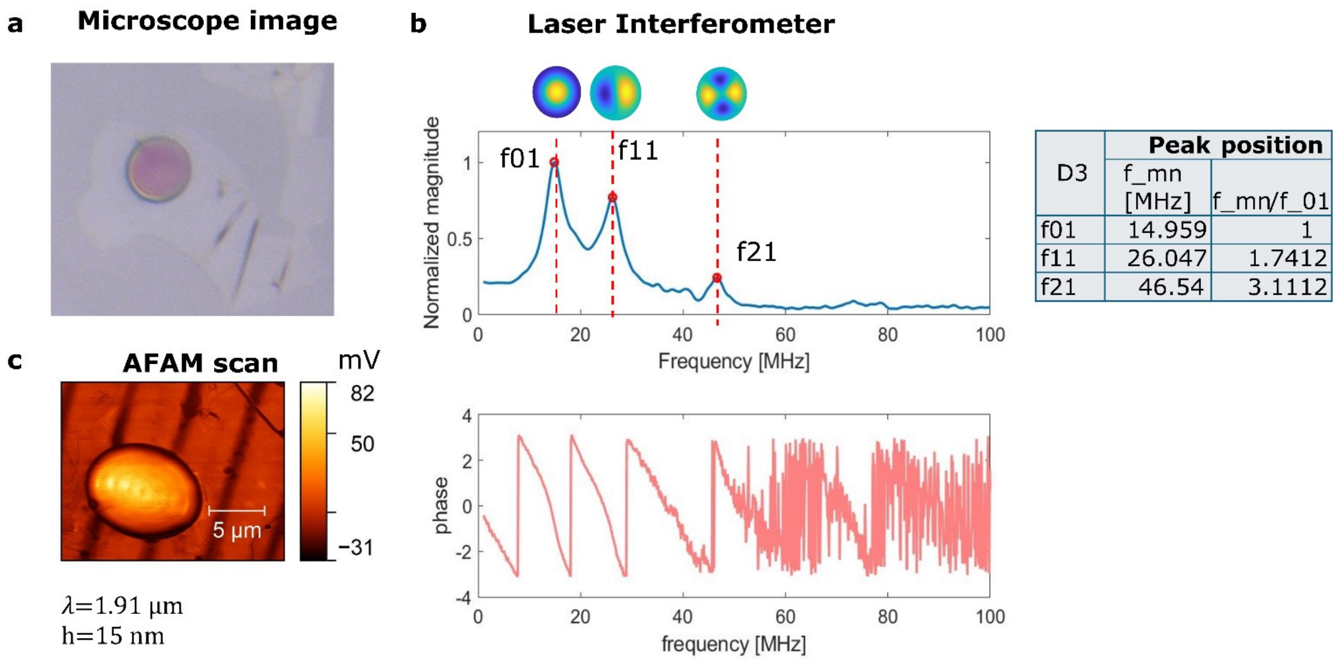

Figure S2.3. Data for Device 3. a) Microscope image, b) Laser interferometer measurements, c) AFAM scan.

S2.4 Device data: D4

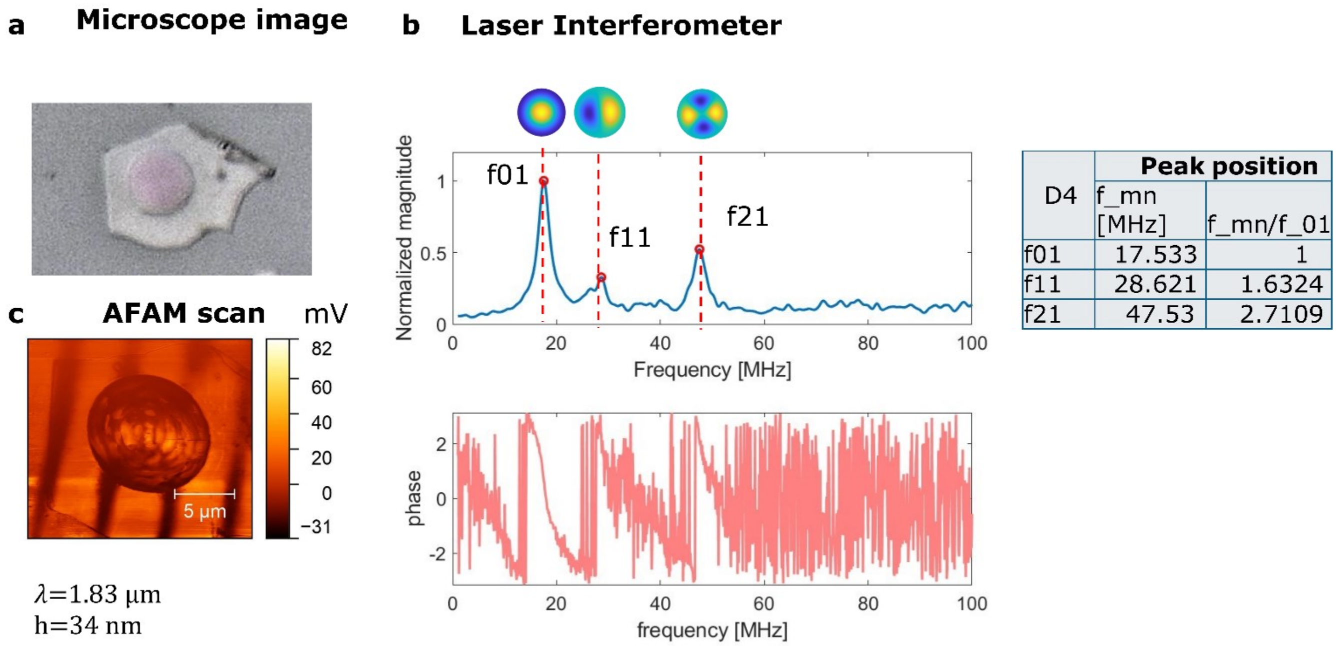

Figure S2.4. Data for Device 4. a) Microscope image, b) Laser interferometer measurements, c) AFAM scan.

S2.5 Device data: D5

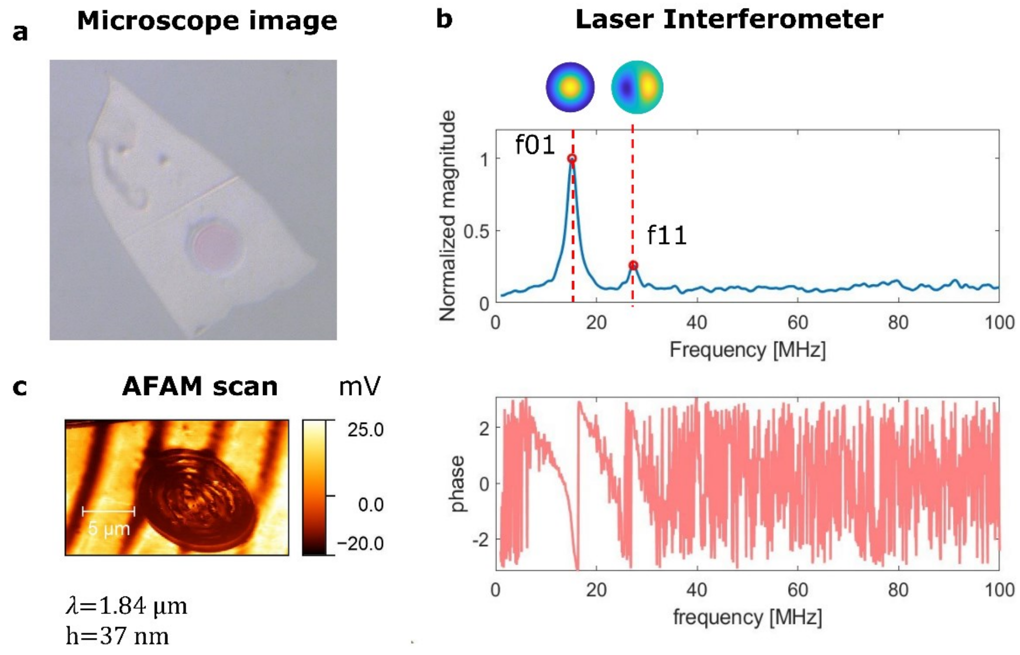

D5	Peak position	
	f_mn [MHz]	f_mn/f_01
f01	15.157	1
f11	27.433	1.8099
f21	-	-

Figure S2.5. Data for Device 5. a) Microscope image, b) Laser interferometer measurements, c) AFAM scan.

S3 AFAM Profiles

The Gwyddion software is used for extracting profiles from vibration data. Multiple (4-6) profiles are acquired along the wave propagation direction with slight variations. Data along each profile line is processed and the distance between consecutive peaks (p) is determined with a peak finding algorithm in MATLAB after smoothing (Figure S3). The peak distance p is converted into a wavelength by $\lambda = 2p$ and to a wavenumber by $k = \pi/p$. Mean and standard deviation of the wavelength (μ_λ , σ_λ) and wavenumber (μ_k , σ_k) are directly calculated from corresponding dataset (Table S3).

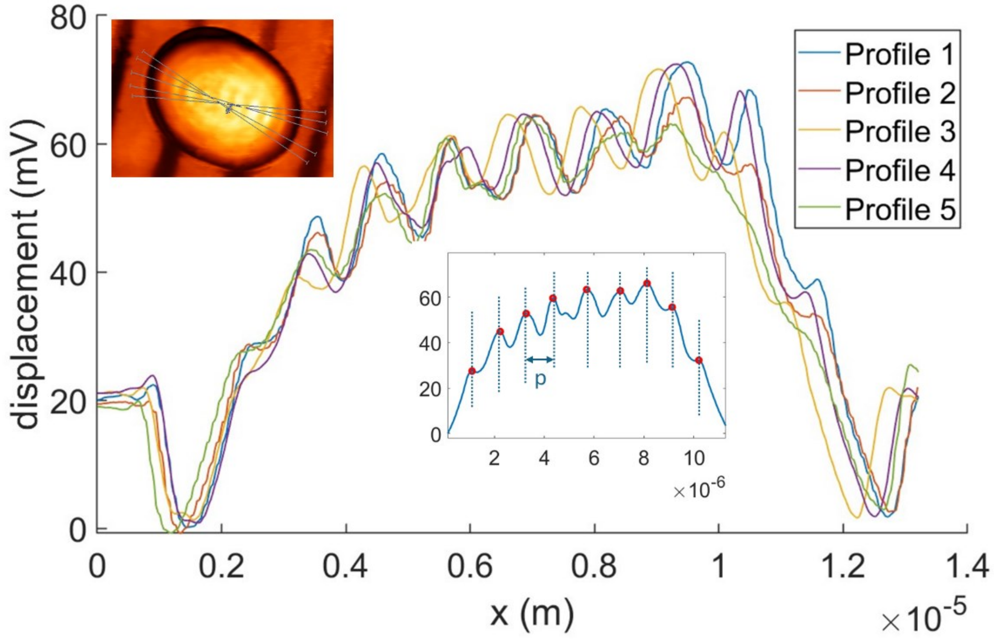

Figure S3. Example of AFAM profile data processing for D2. 5 profiles were taken from the device. Line positions and peak finding on one of the profiles are given as insets.

Table S3 Mean and standard deviation for wavelength and wavenumbers for each device

Device	μ_λ (μm)	σ_λ (μm)	μ_k (μm^{-1})	σ_k (μm^{-1})
D1	2.16	0.49	3.03	0.59
D2	2.28	0.33	2.81	0.41
D3	1.91	0.64	3.87	1.82
D4	1.83	0.49	3.71	1.17
D5	1.84	0.71	3.78	1.12

S4 Dispersion curve fit

$$f = \frac{1}{2\pi}\sqrt{\left(\frac{D}{\rho h}\right)_{eff} k^4 + \left(\frac{T}{\rho h}\right)_{eff} k^2}$$

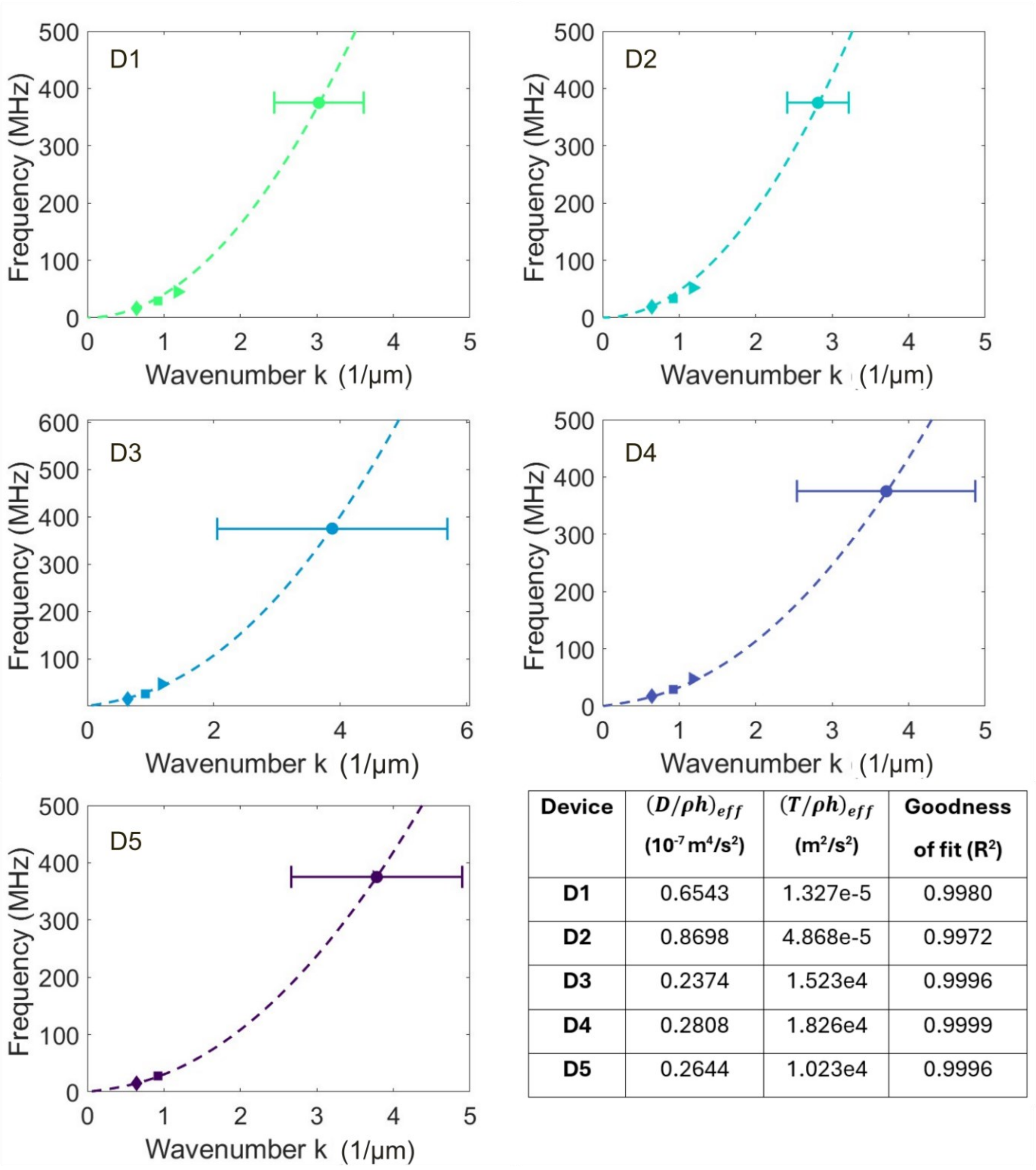

Device	$(D/\rho h)_{eff}$ (10^{-7} m^4/s^2)	$(T/\rho h)_{eff}$ (m^2/s^2)	Goodness of fit (R^2)
D1	0.6543	1.327e-5	0.9980
D2	0.8698	4.868e-5	0.9972
D3	0.2374	1.523e4	0.9996
D4	0.2808	1.826e4	0.9999
D5	0.2644	1.023e4	0.9996

Figure S4.1. Fitted dispersion curves and parameters.

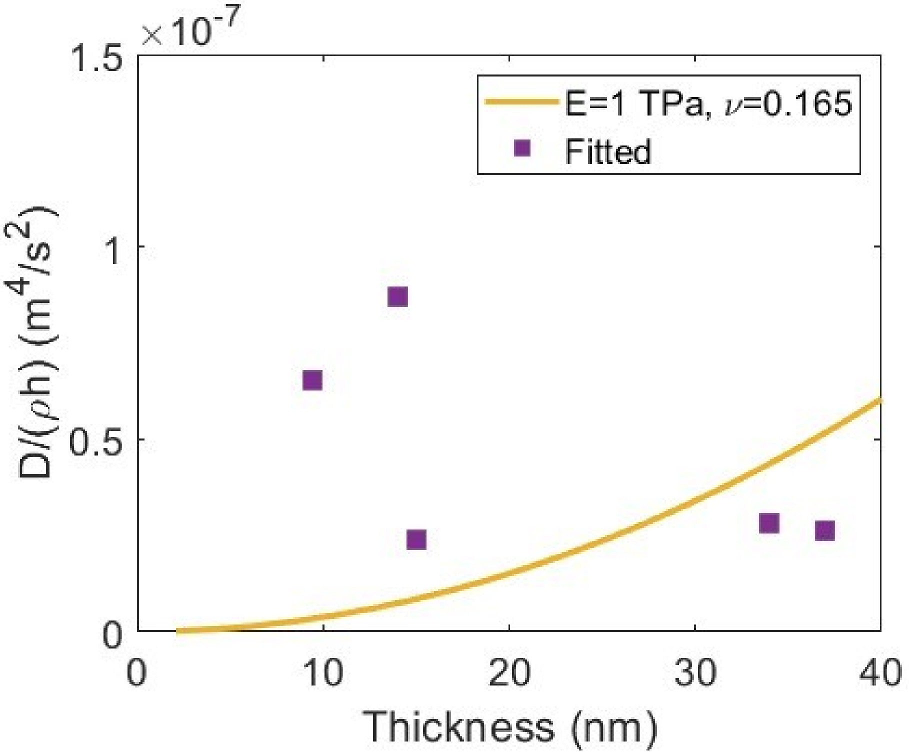

Figure S4.2. Comparison of fitted bending rigidity parameter to analytical estimation with given Young's modulus and Poisson's ratio.

S5 COMSOL Simulation

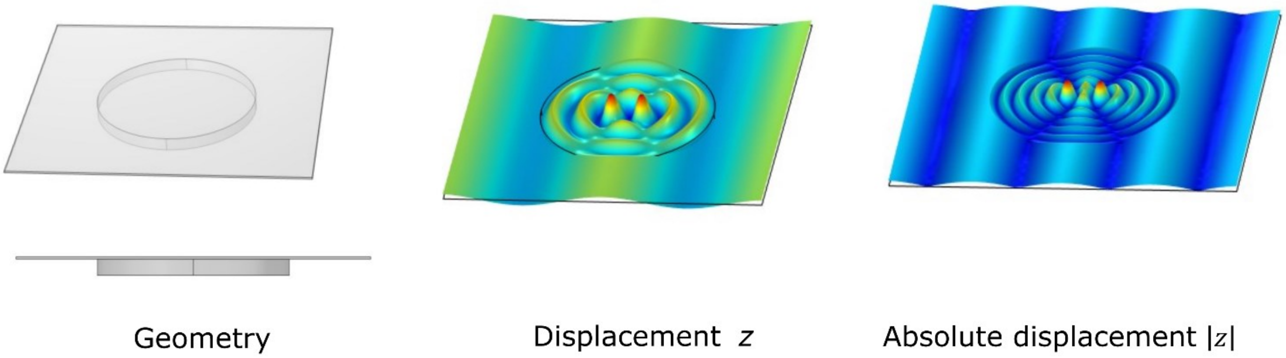

Figure S5. COMSOL model geometry and results for device D4.

The 3D COMSOL model was built to model the vibration of multilayer graphene suspended over a cylindrical cavity filled with air. Graphene was modelled as a shell with an initial tension. Tension and material elastic modulus were determined from fitting results of effective bending rigidity and tension. Domains are modelled as isotropic linear elastic materials and no damping was defined in the model. To represent SAW actuation, a displacement field of 10 µm wavelength and 1 nm amplitude was applied on the supported graphene domain. A frequency domain study was run at 375 MHz.

S6 Additional cavity geometries

By following the same fabrication procedure, additional cavity geometries were also tested. 5x5 µm squares and circular cavities with different diameters (5 µm, 4 µm, 3 µm) were fabricated on LN substrate with thin Cr layer on top, via FIB milling. A graphene flake of 23 nm thickness was stamped on top of the cavities. The SAW device was actuated at the same conditions (375 MHz, 15 dBm) and the resulting vibration field on supported and suspended graphene layers were mapped via AFAM. Clear patterns were observed on suspended parts with smoother surface (*e.g.*, without any particles, wrinkles etc.). For COMSOL simulations, Young's modulus of graphene was taken as 1 TPa and no pretension was applied.

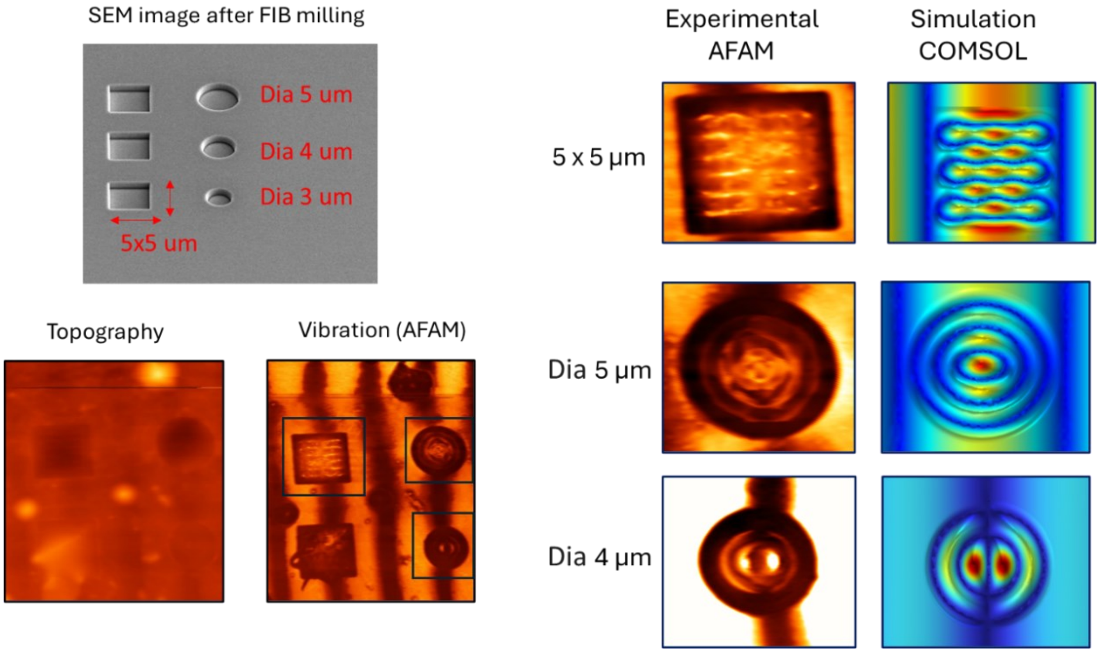

Figure S6. Additional cavity geometries, AFAM scans and COMSOL simulation results.

S7 LDV vs AFAM for mapping suspended graphene vibrations

A suspended graphene structure located in a delay line of a SAW device and the vibration field in that portion is mapped using laser doppler vibrometry (LDV) and atomic force acoustic microscopy (AFAM). The optical image of the 110 nm thick flake suspended over 10 μm cavity is given in Figure S4.1a. The vibration field maps obtained via LDV and AFAM are given in Figure S4.1b and S4.1c, respectively. While the SAW waves at 375 MHz can be mapped with both methods, the spatial resolution of the LDV (~2 μm) does not allow capturing small wavelength (λ~2.5 μm) vibrations on the suspended graphene part, which can be mapped via AFAM with high spatial resolution.

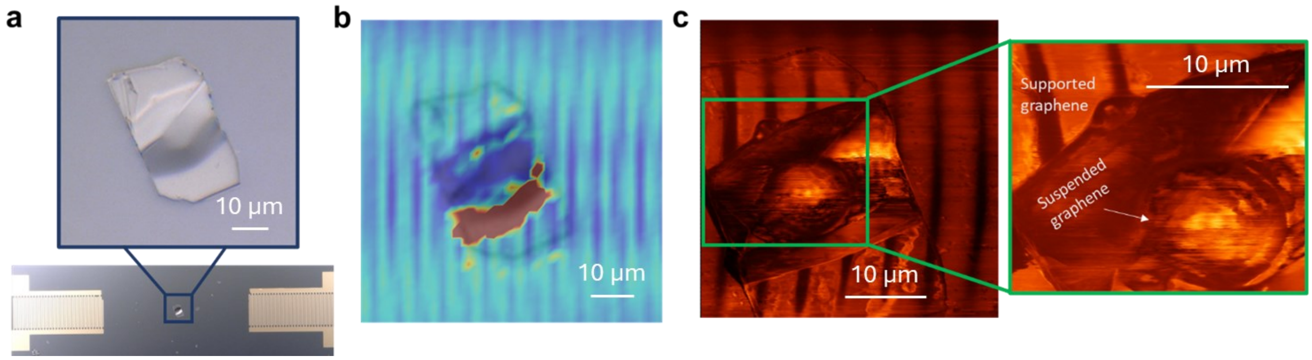

Figure S7. a) Optical image of the graphene layer on SAW delay line, b) LDV measurement of the acoustic wavefield, c) AFAM measurement of the wavefield.

S8.1 AFM topography scans across features

AFM scans showing the topography of the uncovered microcavities and suspended graphene structures are provided in Figure S8.1 below. In panel (a), a microcavity milled by femtosecond laser (fsLaser) is given and panel (b) shows a focused ion beam (FIB) milled cavity. The comparison of the profiles taken over two microcavities is given in (c), showing the FIB leaves a smoother surface around the microcavity edge, and allows better attachment for 2D materials. Panel (d) shows both covered and uncovered microcavities as well as the flake of 23 nm thickness attached on top of microcavities of square (Figure S8.1e), circular and rectangular shapes. In Figure S8.1e, the topography scans of all 5 devices (D1-D5) are provided. From the scans, edges of the cavities underneath the flake and the suspended features can be seen.

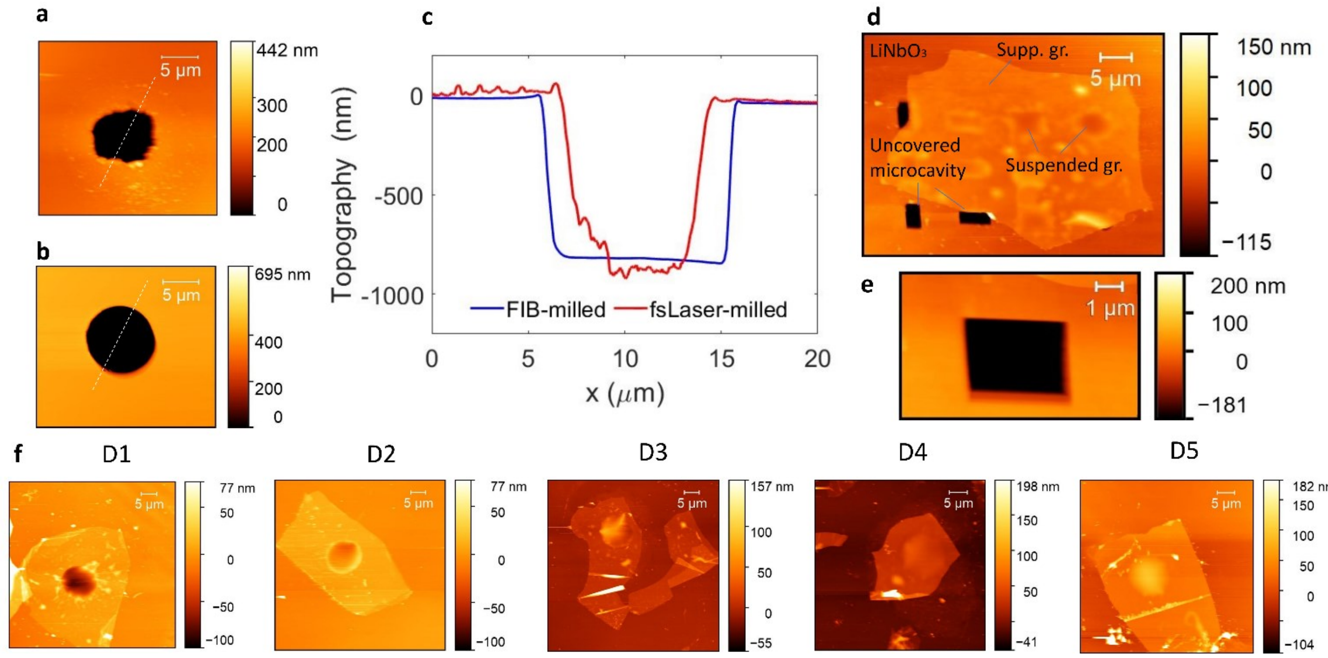

Figure S8.1. AFM topography scans across features; **(a)** fsLaser-milled microcavity,**(b)** FIB-milled microcavity **(c)** comparison of the profiles of a and b, **(d)** 23 nm flake positioned on top of rectangular, square and circular microcavities on $LiNbO_3$ substrate by viscoelastic stamping method described above **(e)** uncovered square microcavity, **(f)** Graphene flakes positioned on top of 10 µm diameter circular microcavities (D1-D5).

S8.2 2D material transfer with PDMS dome stamp

Mechanically exfoliated flakes are first picked up by a PDMS dome stamp. The PDMS dome is then aligned under microscope with the exfoliated flake on Si/SiO_2 surface (Fig. S8.2a). By putting the tip of the PDMS dome in contact with the flake on the Si/SiO_2, the flake is picked up by the stamp. After that, the substrate is changed to the $LiNbO_3$ with the microcavities. Analogously, the flake on the PDMS dome is aligned with the microcavity under microscope, before contact. When the alignment is done, the PDMS/flake stack is put in contact with the cavity/$LiNbO_3$ (Fig. S8.2b). Finally, the stamp is retracted,

leaving the flake on the designated location over the microcavities (Fig. S8.2c). A schematic for transfer with PDMS dome is given in panel d of Figure S8.2.

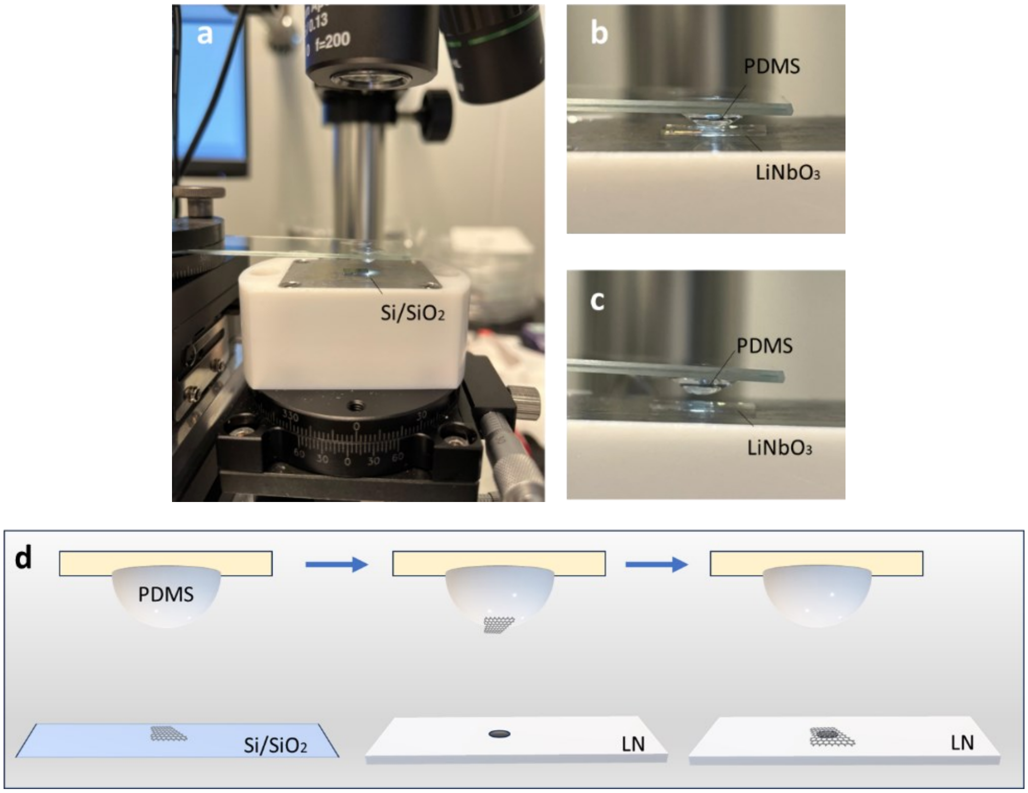

Figure S8.2. 2D material transfer setup: (a) PDMS dome alignment with the flake on Si/SiO_2 substrate, (b) PDMS dome aligned and put into contact with the designated microcavity area on lithium niobate (LN), (c) PDMS stamp is retracted leaving the flake on $LiNbO_3$. (d) Schematic showing the 2DM transfer with PDMS dome.
